# Practical aspects of genetic identification of hallucinogenic and other poisonous mushrooms for clinical and forensic purposes

**DOI:** 10.3325/cmj.2015.56.32

**Published:** 2015-02

**Authors:** Marek Kowalczyk, Andrzej Sekuła, Piotr Mleczko, Zofia Olszowy, Anna Kujawa, Szymon Zubek, Tomasz Kupiec

**Affiliations:** 1 Institute of Forensic Research, Kraków, Poland; 2 Institute of Botany, Jagiellonian University, Kraków, Poland; 3 Institute of Occupational Medicine and Environmental Health, Sosnowiec, Poland; 4 Institute for Agricultural and Forest Environment PAS, Research Station, Turew, Poland; 5 Department of Cosmetology, University of Information Technology and Management, Rzeszów, Poland

## Abstract

**Aim:**

To assess the usefulness of a DNA-based method for identifying mushroom species for application in forensic laboratory practice.

**Methods:**

Two hundred twenty-one samples of clinical forensic material (dried mushrooms, food remains, stomach contents, feces, etc) were analyzed. ITS2 region of nuclear ribosomal DNA (nrDNA) was sequenced and the sequences were compared with reference sequences collected from the National Center for Biotechnology Information gene bank (GenBank). Sporological identification of mushrooms was also performed for 57 samples of clinical material.

**Results:**

Of 221 samples, positive sequencing results were obtained for 152 (69%). The highest percentage of positive results was obtained for samples of dried mushrooms (96%) and food remains (91%). Comparison with GenBank sequences enabled identification of all samples at least at the genus level. Most samples (90%) were identified at the level of species or a group of closely related species. Sporological and molecular identification were consistent at the level of species or genus for 30% of analyzed samples.

**Conclusion:**

Molecular analysis identified a larger number of species than sporological method. It proved to be suitable for analysis of evidential material (dried hallucinogenic mushrooms) in forensic genetic laboratories as well as to complement classical methods in the analysis of clinical material.

Gathering and eating wild mushrooms is an important part of culinary culture in Poland. The popularity of this pastime, coupled with an inability of consumers to distinguish edible from inedible species, results in many cases of poisoning every year (1-5). Poisonous mushrooms can be divided into 9 basic groups based on their type of toxic action (6). Poisoning by gastro-enterotoxic mushrooms, which causes irritation of the gastrointestinal tract (nausea, vomiting, diarrhea), constitutes a relatively minor threat to life and health. The most serious poisonings are caused by mushrooms with cytotoxic-hepatotoxic action, eg, *Amanita phalloides* and the similar species, *Amanita verna*, and *Amanita virosa*, which are often confused with edible species from the genera *Russula*, *Agaricus,* and *Macrolepiota*. Besides *Amanita*, there are highly toxic species belonging to other genera (eg, *Galerina marginata*, *Galerina autumnalis,* and *Lepiota helveola*). In recent years, there has been an increased interest in hallucinogenic mushrooms due to easy access to this source of narcotic substances (7). Possession, sale, and purchase of mushrooms containing narcotic substances is a crime under current law in many countries, including Poland. Thus, identification of mushrooms containing prohibited active substances is of key significance for judicial proceedings. Currently, there are over 200 known species of mushrooms with narcotic properties. The majority of these are species of *Psilocybe*, *Gymnopilus*, and *Paneolus*, as well as some species of toadstools, eg, *Amanita muscaria* and *Amanita pantherina*. Some species from the genera *Conocybe*, *Inocybe*, *Stropharia*, *Pluteus, Hypholoma,* and *Panaeolina* (8,9) are also suspected of containing hallucinogenic substances. The most important psychotropic substances in hallucinogenic mushrooms are psilocybin, psilocin, and baeocystin (8,10). There are occasional fatal cases of poisoning by mushrooms from the genus *Galerina*, mistaken for hallucinogenic mushrooms from the genus *Psilocybe*. Similar mistakes have been made with mushrooms of the genus *Conocybe*, eg, *Conocybe blatteria*, which can be confused with *Psilocybe semilanceata* (11).

In mushroom poisonings, the source of the toxic substance is usually identified using morphological (mainly sporological) and biochemical (toxicological) analysis. Clinical material such as vomit, gastric contents, stool samples, and remnants of uneaten food often contain entire fragments of sporocarps, which can be examined macroscopically and microscopically. This kind of analysis often enables identification of fungal material at the genus level. Sporological analysis, based on microscopic examination of the clinical material, determines the presence of spores and identifies them through comparison with standard fungal spores. In doubtful cases, the result requires verification by 2 or 3 competent laboratory workers (12).

Methods applied in toxicological analysis include high-performance liquid chromatography with diode array detection, gas chromatography with mass spectrometry, and liquid chromatography with mass detection (13,14). These methods enable not only identification of a specific active substance, but also quantitative analysis, provided appropriate standards are used. A difficulty associated with instrumental methods is the need to extract toxic substances from biological material. For amanitine, the extraction method requires the use of immunoaffinity techniques using specific antibodies (15).

Fungal species identification based on genetic analysis is a promising alternative to morphological and physicochemical studies. Several polymerase chain reaction (PCR)-based methods of DNA analysis have been employed in species identification, eg, random amplification of polymorphic DNA (RAPD), amplified fragment length polymorphism (AFLP), restriction fragment length polymorphism (RFLP), and real-time PCR and DNA sequencing. Some of these methods, such as RAPD and AFLP methods, analyze the whole genome and are applied in the identification of polymorphisms and determination of genetic distance between species, but are not suitable for identification of closely-related species (16,17). Other methods, eg, real-time PCR and DNA sequencing, are based on the amplification of DNA-fragments of taxonomic relevance. In animals, the region encoding the enzyme cytochrome oxidase (COI) has been proposed as a target region in DNA barcoding, enabling identification of 95% of species (18,19), but in fungi, this region enables identification of only 20%-70% of species (19). In fungi, the most important target region is nuclear DNA encoding ribosomal RNA (nrDNA), arranged in the form of tandem repeats. The number of copies of nrDNA genes in mushrooms varies from 60 to 220, depending on the species. Fragments encoding ribosomal RNA are separated by non-coding fragments, which are removed after the transcription phase. These so-called internal transcribed spacers (ITS1 and ITS2) and non-transcribed intergenic spacers (IGS) are characterized by the presence of polymorphisms of length and sequence, which make them highly useful for species identification.

There are primers serving in the amplification of both ITS regions, including the 5,8S coding region, as well as species-specific primers targeting the ITS (20,21). Lee et al (22) showed that hallucinogenic mushrooms from the genera *Panaeolus* and *Psilocybe* can be differentiated on the basis of differences in the lengths of ITS1 PCR products. However, for some species, sequencing of the studied fragments is necessary. Knowing the entire sequence of the studied region also enables better differentiation of analyzed samples. As numerous studies have shown (22-26), analysis of the sequences of ITS regions is useful for creating molecular systematics at the species level, and even for defining intraspecific geographic differentiation. Furthermore, existence of numerous copies of these regions enables their amplification even in considerably degraded material, which makes them very attractive for forensic testing (22). Studies conducted on the polymorphism of the ITS region, encompassing mushrooms belonging to many taxa, have shown the usefulness of these regions as a basic universal marker in the Fungi kingdom (27-29). The aim of this study was to develop, optimize, and assess the usefulness of a molecular method based on sequencing of the ITS region of nrDNA in the forensic and clinical identification of mushrooms. Our research is the first with such a large number of samples of clinical and forensic material originating from Central Europe.

## Material and methods

### Analyzed material

The study was carried out in the period 2011-2013. We used clinical forensic samples containing fragments of mushrooms, such as vomit, gastric contents, food remains, and shredded dried mushrooms. Samples of clinical material, as well as some food remains, were supplied by the Institute of Occupational Medicine and Environmental Health in Sosnowiec. Some of the dried mushrooms came from the Department of Toxicology of the Institute of Forensic Research (5 samples). We prepared the remaining samples of dried mushrooms and food ourselves (by subjecting sporocarps of fungi of various edible, inedible, and poisonous species to heat treatment or drying). A total of 221 samples were studied, including 132 samples of clinical material, 65 dish samples, and 24 samples of dried mushrooms. Clinical material consisted of gastrointestinal content and feces, with and without visible mushroom fragments. Bits that looked like fragments of fruiting bodies (sporocarps) of mushrooms were analyzed and when no obvious fragments were present (eg, in some samples of stomach contents obtained by gastric lavage), all the solid material obtained following centrifugation of the sample was collected. Samples thus obtained were twice rinsed in 70% ethanol and deionized water prior to DNA extraction.

### DNA extraction and amplification

Based on a preliminary study on various DNA extraction methods (data not shown), we used the magnetic method, employing a BioRobot M48 robotic workstation (Qiagen, Hilden, Germany) and compatible MagAttract reagent kits (Qiagen) for DNA isolation, as this proved the most efficient. The procedure followed the protocols provided by Qiagen (*http://www.qiagen.com/pl/products/catalog/sample-technologies/dna-sample-technologies/genomic-dna/magattract-dna-mini-m48-kit/#resources*). This method, as well as methods employing similar reagent kits, are routinely used in forensic laboratory practice in the process of DNA isolation from biological traces (30-33).

PCR reactions were performed using HotStarTaq Master Mix Kit (Qiagen) in an ABI 9700 thermocycler (Applied Biosystems, Foster City, USA). Primers complementary to the ITS1 region (primers ITS1F and ITS2) and ITS2 region (primers ITS3 and ITS4) were initially used for amplification (20,21). Analysis of samples originating from gastrointestinal contents yielded either sequences of sac fungi (Ascomycota) colonizing the gastrointestinal tract (most frequently *Candida* spp) or a mixture of products unsuitable for further analysis. The vast majority of edible mushrooms and poisonous mushrooms belong to the phylum Basidiomycota. Accordingly, pairs of primers ITS3 (5′GCATCGATGAAGAACGCAGC3′) (20) and ITS4B (5′CAGGAGACTTGTACACGGTCCA3′) (21), which amplify the ITS2 region, along with a fragment of the region encoding 28S nrDNA, were finally used for DNA amplification from clinical forensic samples. Primer ITS4B (Basidiomycota-specific) was applied in order to obtain an amplification product of Basidiomycota DNA exclusively. PCR reactions were carried out using the following thermal profile: 15 min at 95°C, followed by 35 cycles of 30 sec at 95°C, 30 sec at 58°C, 1 minute at 72°C, and termination 10 min at 72°C. In order to check the efficiency of amplification, electrophoretic separation was performed with a QIAxcel apparatus for automated capillary electrophoresis using a QIAxcel DNA Screening Kit (Qiagen). For purification of amplification products, the enzymatic method was applied, using reagents Exonuclease I (ExoI) and Thermo Scientific FastAP Thermosensitive Alkaline Phosphatase (FastAP) (Thermo Scientific, Waltham, USA).

### DNA sequencing and sequence analysis

Sequencing reactions were carried out in both directions, using amplification primers and a BigDye Terminator Cycle Sequencing kit, v. 3.1 (Applied Biosystems), according to the manufacturer’s recommendations (25 cycles of 10 min at 96°C, 5 sec at 55°C, and 2 min at 60°C). The products were purified using ExTerminator 96 plates (A&A Biotechnology, Gdynia, Poland) and subjected to electrophoretic separation, with the application of ABI Prism 3130xl genetic analyzer (Applied Biosystems) (gel POP 7, capillary 36 cm). The obtained sequences were aligned using Clustal W, v. 2.0.12 (34) contained in BioEdit, v. 7.0.8.0 (35), and DNA Baser Sequence Assembler, v. 4.10.1.13 (36).

### Comparison with the NCBI database

For the purpose of species identification, the obtained sequences were compared with sequences available in the National Center for Biotechnology Information (NCBI) internet database (GenBank), using the BLAST program (37). Depending on the level of consistency with the reference sequences, six identification levels (Table 1) were determined (38).

**Table 1:** Levels of sample identification determined for the present study

Level of identification	Level of sequence consistency with National Center for Biotechnology Information gene bank database
**Species**	Maximal consistency 97% or greater, with reference sequences of single species.
**Group of species**	Maximal consistency 97% or greater, with reference sequences of several different species that are closely related to each other.
**Genus**	1. Maximal consistency below 97%, with reference sequences of one species or several species belonging to the same genus. 2. Maximal consistency 97% or greater, with reference sequences of several different species that are not closely related to each other, but belong to the same genus.
**Group of genera**	Sequences of the highest consistency belong to members of several genera that are closely related to each other.
**Family**	Sequences of the highest consistency belong to members of several genera of to the same family.
**No identification**	Sequences of the highest consistency belong to members of different families.

### Toxicological analysis

Results of toxicological analysis were available for 5 samples of dried mushrooms. This analysis was carried out using gas chromatography methods coupled with mass spectrometry and high-performance liquid chromatography, aimed at determining the content of psychoactive substances (data obtained from the Department of Toxicology of the Institute of Forensic Research).

### Sporological analysis

Results of sporological analysis were available for 57 clinical samples (data obtained from the Institute of Occupational Medicine and Environmental Health in Sosnowiec). Microscopic analysis included three steps (12):

1. Degreasing, concentrating, and dissolving the analyzed material.

2. Chemical microreactions with Melzer’s reagent, Sudan III, and 10% HCl.

3. Microscopic comparison with standard fungal spores from a collection acquired by the Department of Laboratory Diagnostics, Institute of Occupational Medicine and Environmental Health in Sosnowiec.

## Results

### Results of molecular analysis

Of 221 samples, positive amplification results were obtained for 192 (87%). The percentage of successfully amplified samples depended on the type of studied material, ranging from 75% (15 out of 20 samples) for gastrointestinal contents with no visible fragments of mushrooms to 100% (24 samples) for dried mushrooms (Table 2). Among positively amplified samples, the lowest percentage of sequences suitable for further analysis was obtained for samples of feces (35%, 6 out of 17 samples) and the highest for food remains (91%, 59 out of 65 samples) and dried mushrooms (96%, 23 out of 24 samples) (Table 2). For highly processed material, we obtained mixtures of sequencing products that were unsuitable for interpretation, constituting from 27% to 58% (gastrointestinal content and feces, respectively) of the positively amplified samples.

**Table 2:** Results of amplification and sequencing of the ITS2 region for clinical-forensic samples

Type of material	Number of studied samples	Amplification success, N (%)	Sequencing results		Sequencing success (%)
			readable sequences (N)	mixtures of products (N)	
**Feces with visible fragments of mushrooms**	13	12 (92)	5	7	38
**Feces without visible fragments**	4	2 (50)	1	1	25
**Gastrointestinal content with visible mushroom fragments**	95	79 (83)	53	26	56
**Gastrointestinal content without visible fragments**	20	15 (75)	11	4	55
**Food remains**	65	60 (92)	59	1	91
**Dried mushrooms**	24	24 (100)	23	1	96
**Total**	221	192 (87)	152	40	69

### Comparison with the NCBI database

Comparison with the sequences stored in the NCBI gene bank (GenBank) enabled us to classify 90% of samples for which sequences were obtained at the level of species or a group of closely related species. In 10% of samples, identification was possible at the genus level (Table 3).

**Table 3:** Results of molecular and sporological identification of forensic-medical samples

Level of identification	Molecular identification, N (%)	Sporological identification, N (%)
**Species**	129 (85)	30 (53)
**Group of species**	7 (5)	0 (0)
**Genus/group of genera**	16 (10)	8 (14)
**Family**	0 (0)	5 (9)
**No identification**	0 (0)	14 (25)
**Total**	152 (100)	57 (100)

### *Results of toxicological and sporological analysi***s**

In one of the studied samples for which toxicological data were available, the presence of psilocin was ascertained. Genetic analysis identified this sample (S017) as belonging to the species *Psilocybe semilanceata*. In the 4 remaining samples, the presence of psilocin was not ascertained. However, genetic analysis identified 3 samples (S072, S073, and S074) as *Psilocybe cubensis* and one sample (S016) as a specimen of *Agrocybe pediades* (Supplementary Table)(web extra material 1). Fungal spores were identified in 76% of microscopically examined samples and their analysis enabled us to identify over 50% of samples at the species level (Table 3).

### Comparison of the molecular and sporological method

For 30% of microscopically examined samples, sporological and molecular identification was consistent at least at the genus level, whereas for 26% of samples there was no consistency. For most of the samples with negative sporological identification, the material was identified by molecular methods (Table 4). Sporological analysis revealed the presence of fungi that had not been identified by molecular methods in the following samples (Supplementary Table)(web extra material 1): sample S154 (spores of *Mycena* sp and *Coprinus* sp); sample S053 (spores of *Amanita* sp); sample S200 (spores of *Macrolepiota* sp).

**Table 4:** Concordance between sporological and molecular method

Level of concordance	Samples, N (%)
**The same species**	8 (14)
**The same group of species**	0 (0)
**The same genus/group of genera**	9 (16)
**The same family**	5 (9)
**No concordance**	15 (26)
**No spores present/Molecular identification negative**	1 (2)
**No spores present/Molecular identification positive**	13 (23)
**Molecular identification negative/Sporological identification positive**	6 (11)

## Discussion

The study found that the molecular method for fungi species identification was useful for forensic and clinical purposes. The method yielded a positive result despite the fact that fruiting bodies of fungi were processed to a high degree (and hence DNA was degraded). The best results were obtained for dried mushrooms and food leftovers. However, this method has certain limitations. In clinical samples, there is the problem of DNA mixtures, resulting in unreadable sequencing results. The occurrence of mixtures of sequences may be linked to the presence of other species of fungi in the gastrointestinal tract or to contamination by molds during sample storage prior to analysis. Since most fungi colonizing the human digestive tract (eg, *Candida albicans*), as well as molds, belong to the phylum Ascomycota, and a great majority of edible and poisonous mushrooms belong to Basidiomycota, this problem can be partially solved by applying primers specific for the latter (eg, ITS4B). However, this limits the analysis to the ITS2 region due to a lack of primers that are both specific for Basidiomycota and enable amplification of the ITS1 region. Application of a primer specific for Basidiomycota results in the impossibility of identifying poisonous mushrooms belonging to Ascomycota (eg, members of the genus *Gyromitra*). On the other hand, the possible (though rare) presence of microscopic fungi belonging to Basidiomycota cannot be eliminated when using such an amplification primer. One example might be a sequence of a member of the genus *Kwoniella* found in the material collected from the gastrointestinal contents of patient No. 55. Genus *Kwoniella* encompasses the five currently known species of fungi from the order Tremellales, which are closely related to the genus *Filobasidiella* and consist of saprobiontic, microscopic fungi that do not form fruiting bodies (39-43). The presence of one of the *Kwoniella* species was ascertained in the gastrointestinal tract (43). There is also the problem of mixtures of various species of mushrooms consumed together, which are often impossible to be separated due to significant fragmentation and degradation of fungal material in gastrointestinal contents.

One solution to the problem of mixtures might be the application of next generation sequencing. However, this method is still too costly for routine forensic and clinical analyses. Other possible solutions include the use of species-specific primers for amplification or methods based on specific oligonucleotide probes. To identify the hallucinogenic mushroom *Psilocybe semilanceata,* Adamczyk et al (44) applied PCR primers enabling specific amplification of the ITS1 region of this species. Harper et al (45) designed a method using macroarrays with oligonucleotide probes based on species-specific fragments of ITS region. This method rapidly identifies the most toxic mushrooms from the genus *Amanita*. Harper et al showed that this method can be used to distinguish individual species in samples containing mixtures of mushroom fragments from different species. However, in some cases false positive and false negative reactions occurred. This method, though fast and relatively inexpensive, requires development of oligonucleotide probes for additional species of toxic and hallucinogenic mushrooms. Thus, to obtain information on the present polymorphism needed to create oligonucleotide probes it is necessary to sequence the ITS region.

The most important stage of our method is the comparison of obtained sequences with reference sequences available in databases. This approach has been applied in studies on hallucinogenic mushrooms present on the Japanese market (7). The obtained sequences were matched using BLAST software with sequences of ITS regions in several databases (GenBank, EMBL, and DDBJ). This enabled species identification of all samples (7). The limitation of this approach is that publicly available databases may contain mistakes resulting from incorrect species identification or low quality of sequences (23,46,47). For example, in our study a high degree of consistency was found between all sample sequences correctly identified as *Amanita muscaria* and an individual sequence of *Amanita gemmata* from the NCBI database (Supplementary Table(web extra material 1), samples: S131, S186-S188, S195, S197, S202-S205, S209, S217). One solution to this problem might be to create reference databases containing material from a defined geographical region that has been precisely and reliably identified.

Our research showed that sequencing of the ITS2 fragment was sufficient in most cases and that it correctly identified the most frequent causes of mushroom poisonings, as well as the most common hallucinogenic mushrooms. Among the 152 studied samples for which correct sequences of the ITS region were obtained, the great majority of poisonous and hallucinogenic mushrooms were identified at the species level (Supplementary Table(web extra material 1)). Identification problems (ie, identification only at the genus level) were limited to certain members of the genera *Russula* (samples S136, S137, S166, S167, and S192) and *Cortinarius* (sample S042). Within *Russula,* however, there are no highly poisonous species, only those characterized by gastroenterotoxic action. Genus *Cortinarius* is more problematic, as it includes highly poisonous species. Species identification is also necessary in the case of hallucinogenic mushrooms from the genus *Psilocybe*, as it includes both species that produce toxic substances and species that are not toxic (8,9,11,48).

Other studies show that the high degree of polymorphism of sequences of the ITS region may lead to ambiguous identifications of closely related species (49,50). Nugent and Saville (51) also used sequencing of the region encoding the nuclear large ribosomal subunit of rRNA (nLSU rRNA) with a lower degree of polymorphism, which highly reliably differentiated closely related species of hallucinogenic mushrooms from North America.

For certain groups of fungi, additional sequencing of the nLSU region may not suffice for correct species differentiation. In some species of genus *Cortinarius* that are difficult to differentiate using the ITS and nLSU regions, full identification was performed only by analysis of sequences of genes encoding RNA polymerase (RPB1 and RPB2) in combination with sequences of the ITS region (50). Accordingly, when identification based on analysis of the ITS region (eg, of a member of the genus *Cortinarius*) is doubtful, it would be advisable to use additional markers (eg, LSU, RPB1, and RPB2). This, however, may be difficult, as sequences of these regions for many species are often unavailable in reference databases.

Kallifatidis et al (52) used a method based on the analysis of fluorescent random amplified microsatelites to distinguish between closely related species that cannot be differentiated using sequencing of the ITS region (eg, *Psilocybe moravica*, *P. serbica,* and *P. bohemica*). Using this method, a high degree of intraspecific variation can also be observed. The authors examined 20 species belonging to the *Amanita* and *Psilocybe* genera. Application of this method in forensic or clinical practice requires development of a reference database including the most common hallucinogenic and other toxic mushrooms. Also, the analysis of clinical samples may be significantly complicated by the presence of gastrointestinal microbiota. Our results showed that molecular analysis identified a larger number of mushroom species in clinical forensic samples than sporological and toxicological methods. In some samples, sporological analysis revealed the presence of species not identified by molecular method. However, genetic identification significantly increased the number of identified species (eg, in samples taken from patients No. 12, 13, 56, 65, 67, 75, and 81, Supplementary Table(web extra material 1)).

Sporological methods were less precise than molecular methods, due to the similarity of spores of various genera/species of mushrooms (eg, in the case of patients No. 12 – *Cortinarius*/*Inocybe*, 22 – *Lactarius*/*Russula*, 53 – *Stropharia*/*Macrolepiota*, 67 – *Chlorophyllum*/*Macrolepiota*) or the impossibility to identify spores more specifically than at the genus level. Morphological features of spores are sometimes similar in poisonous and non-toxic species (53). In a microscopic preparation, *Amanita* spores, including *A. phalloides* spores, may be confused with leukocytes, erythrocytes, epithelial cells, yeast cells, or fat droplets, which abound in the gastrointestinal tract (12).

Inappropriate conditions (eg, high humidity and temperature, exposure to light) and long storage of the studied material frequently hinder toxicological analysis and may lead to total breakdown of certain toxic substances. Examples of substances that are sensitive to storage conditions are psilocin and psilocybin, which break down readily in mushrooms stored at room temperature, as well as in aqueous solutions of low concentration (13,54,55). This is confirmed by the results of our research. Genetic analysis classified three samples of dried mushrooms in which toxicological analysis failed to reveal the presence of psilocin within the species *Psilocybe cubensis*.

Our results show that molecular method is suitable for use in forensic genetic laboratories and that it can be implemented using equipment and reagents employed in such laboratories. The advantage of this method over most other genetic methods used in toxic mushrooms analysis is that it can identify a very broad range of fungal species. However, in clinical poisonings, where rapid identification of the source of poisoning is paramount, this method can complement classical analytical methods, but cannot fully replace them.
