# Supplementary material for: Practical aspects of genetic identification of hallucinogenic and other poisonous mushrooms for clinical and forensic purposes
Source: Croat Med J. 2015 Feb;56(1):32–40. doi: 10.3325/cmj.2015.56.32 (PMC4364345; doi:10.3325/cmj.2015.56.32)
Supplement: Supplementary Table 1 [file CroatMedJ_56_s001.pdf]

Sample number	Source of sample	Type of material	Genetic identification - NCBI database	Match with database	Number of consistent sequences	Accession number of most consistent sequence	Identification level	Microscopic identification	Identification level
S016	Dried mushrooms 01	Dried mushrooms	*Agrocybe pediades*	99-100%	>5	JN684779.1	species[1]	not analyzed	unidentified
S017	Dried mushrooms 02	Dried mushrooms	*Psilocybe semilanceata*	99-100%	>5	HM035080.1	species[2]	not analyzed	unidentified
S018	Dried mushrooms 03	Dried mushrooms	*Amanita citrina*	98-99%	3<br>4	JN235140.1<br>FJ210727.1	species	not analyzed	unidentified
S019	Dried mushrooms 04	Dried mushrooms	*Agaricus bisporus*	99%	3	AY484692.1	species[3]	not analyzed	unidentified
S020	Dried mushrooms 05	Dried mushrooms	*Cantharellus tubaeformis*	99%	>5	AF385633.1	species	not analyzed	unidentified
S022	Dried mushrooms 07	Dried mushrooms	*Craterellus cornucopioides*	99%	1	JF907967.1	species	not analyzed	unidentified
S023	Dried mushrooms 08	Dried mushrooms	*Flammulina elastica*<br>*F. velutipes*<br>*F.rossica*	99-100%	4<br>1<br>1	EU191055.1<br>JX294499.1<br>EU191054.1	species group	not analyzed	unidentified
S024	Dried mushrooms 09	Dried mushrooms	*Laccaria amethystina*	99%	>5	HM189775.1 (ITS1) AF539737.1 (ITS2)	species	not analyzed	unidentified
S025	Dried mushrooms 10	Dried mushrooms	*Lactarius lignyotus*	99-100%	>5	JQ446117.1	species[4]	not analyzed	unidentified
S026	Dried mushrooms 11	Dried mushrooms	*Lactarius picinus*<br>*L. fuliginosus*	99-100%	>5<br>3	JQ446133.1<br>AY606947.1	species group	not analyzed	unidentified
S027	Dried mushrooms 12	Dried mushrooms	*Lepista nuda*	98%	4	FJ810156.1	species[5]	not analyzed	unidentified
S028	Dried mushrooms 13	Dried mushrooms	*Macrolepiota procera*	99-100%	2	JQ683113.1	species[6]	not analyzed	unidentified
S029	Dried mushrooms 14	Dried mushrooms	*Marasmius alliaceus*	99%	5	JN943597.1	species	not analyzed	unidentified
S030	Dried mushrooms 15	Dried mushrooms	*Volvariella gloicephala*	99%	>5	HM562209.1	species	not analyzed	unidentified
S072	Dried mushrooms 16	Dried mushrooms	*Psilocybe cubensis*	100%	5	HM035082.1	species	not analyzed	unidentified
S073	Dried mushrooms 16	Dried mushrooms	*Psilocybe cubensis*	100%	5	HM035082.1	species	not analyzed	unidentified
S074	Dried mushrooms 16	Dried mushrooms	*Psilocybe cubensis*	100%	5	HM035082.1	species	not analyzed	unidentified
S141	Dried mushrooms 17	Dried mushrooms	*Hypholoma fasciculare*	100%	5	FJ430717.1	species	not analyzed	unidentified
S142	Dried mushrooms 18	Dried mushrooms	*Boletus luridiformis*[7]	99%	3	FM958177.1	species	not analyzed	unidentified
S143	Dried mushrooms 19	Dried mushrooms	*Boletus calopus*	100%	3	HM347645.1	species	not analyzed	unidentified
S144	Dried mushrooms 20	Dried mushrooms	*Boletus calopus*	100%	3	HM347645.1	species	not analyzed	unidentified
S145	Dried mushrooms 21	Dried mushrooms	*Boletus calopus*	100%	3	HM347645.1	species	not analyzed	unidentified
S224	Dried mushrooms 22	Dried mushrooms	*Serpula lacrymans*	100%	>5	AM946629.1	species	not analyzed	unidentified
S052	Patient 14	Faeces with mushroom fragments	*Chlorophyllum brunneum*	99-100%	>5	AY083208.1	species	not analyzed	unidentified
S150	Patient 53	Faeces with mushroom fragments	*Stropharia rugosoannulata*	99%	1	KC176328.1	species	*Macrolepiota procera*	species
S173	Patient 65	Faeces with mushroom fragments	no sequence	-			unidentified	*Russula sp.*	genus
S176	Patient 67	Faeces with mushroom fragments	*Russula vinosa*	99%	3	AF418638.1	species[8]	*Macrolepiota procera*<br>*Boletaceae*	species family

S192	Patient 75	Faeces with mushroom fragments	*Russula spp.*<br>*(max. R. decolorans*<br>*R. cf. vinosa)*	91-94%	>5	FJ845432.1<br>FJ845428.1	genus	*Macrolepiota procera*	species
S206	Patient 83	Faeces with mushroom fragments	no sequence	-			unidentified	*Boletaceae*	family
S213	Patient 81	Faeces with mushroom fragments	*Amanita muscaria*	98%	>5	JF899546.1	species[9]	no spores	unidentified
S123	Patient 51	Faeces without mushroom fragments	*Paxillus involutus*	98-100%		FR750011.1	species	*Macrolepiota procera*	species
S012	Food leftover 01	Food leftovers	*Tremella fuciformis*	97-99%	>5	FJ501580.1	species	not analyzed	unidentified
S031	Food leftover 02	Food leftovers	*Scleroderma areolatum*<br>*S. verrucosum*	99%	5<br>1	FM213352.1<br>EU784416.1	species group	not analyzed	unidentified
S032	Food leftover 03	Food leftovers	*Lactarius deliciosus*	100%	>5	FJ858745.1	species	*Lactarius deliciosus*	species
S033	Food leftover 03	Food leftovers	*Lactarius deliciosus*	100%	>5	FJ858745.1	species	*Lactarius deliciosus*	species
S037	Food leftover 04	Food leftovers	*Inocybe melanopus*	100%	2	AM882727.2	species	*Paxillus involutus*	species
S038	Food leftover 04	Food leftovers	*Inocybe melanopus*	100%	2	AM882727.2	species	*Paxillus involutus*	species
S039	Food leftover 05	Food leftovers	*Xerocomus badius*	100%	>5	HQ207696.1	species	*Boletaceae*	family
S040	Food leftover 05	Food leftovers	*Xerocomus badius*	100%	>5	HQ207696.1	species	*Boletaceae*	family
S041	Food leftover 06	Food leftovers	*Tylopilus felleus*	99-100%	4	HM190016.1	species	*Boletaceae*	family
S043	Food leftover 47	Food leftovers	*Agaricus xanthodermus*	100%	2	AY484689.1	species	*Agaricus sp.*	genus
S044	Food leftover 47	Food leftovers	*Agaricus xanthodermus*	100%	2	AY484689.1	species	*Agaricus sp.*	genus
S058	Food leftover 07	Food leftovers	*Suillus luteus*	100%	5	FJ236030.1	species	not analyzed	unidentified
S059	Food leftover 07	Food leftovers	*Suillus luteus*	100%	5	FJ236030.1	species	not analyzed	unidentified
S060	Food leftover 07	Food leftovers	*Suillus luteus*	100%	5	FJ236030.1	species	not analyzed	unidentified
S061	Food leftover 07	Food leftovers	*Lactarius deliciosus*	100%	>5	FJ858745.1	species	not analyzed	unidentified
S065	Food leftover 08	Food leftovers	*Xerocomus badius*	100%	>5	HQ207696.1	species	not analyzed	unidentified
S067	Food leftover 09	Food leftovers	*Lactarius rufus*	99-100%	>5	DQ097868.1	species	not analyzed	unidentified
S068	Food leftover 09	Food leftovers	*Lactarius rufus*	99-100%	>5	DQ097868.1	species	not analyzed	unidentified
S069	Food leftover 10	Food leftovers	*Agaricus bisporus*	99%	5	AY484692.1	species1	not analyzed	unidentified
S070	Food leftover 11	Food leftovers	*Agaricus bisporus*	99%	5	AY484692.1	species1	not analyzed	unidentified
S075	Food leftover 13	Food leftovers	*Xerocomus badius*	100%	>5	HQ207696.1	species	not analyzed	unidentified
S076	Food leftover 13	Food leftovers	*Agaricus bisporus*	99%	5	AY484692.1	species1	not analyzed	unidentified
S081	Food leftover 48	Food leftovers	*Xerocomus badius*	100%	>5	HQ207696.1	species	not analyzed	unidentified
S086	Food leftover 14	Food leftovers	*Agaricus osecanus10*<br>*A.albolutescens*<br>*A. urinascens var. excellens*	99%	1<br>1<br>1	AY484670.1<br>AY484675.1<br>AY484682.1	species group	not analyzed	unidentified
S102	Food leftover 14	Food leftovers	*Lactarius salmonicolor*<br>*L. deterrimus11*<br>*L. rubrilacteus*<br>*L. sanguifluus*<br>*L. vinosus*	98%	3<br>2<br>1<br>1<br>1	DQ922551.1<br>EF685051.1<br>FJ627032.1<br>JQ685723.1<br>AY953420.1	species group	not analyzed	unidentified

S103	Food leftover 14	Food leftovers	*Lactarius salmonicolor*<br>*L. deterrimus11*<br>*L. rubrilacteus*<br>*L. sanguifluus*	98%	3<br>2<br>1<br>1	DQ922551.1<br>EF685051.1<br>FJ627032.1<br>JQ685723.1	species group	not analyzed	unidentified
S109	Food leftover 15	Food leftovers	*Sullius luteus*	99%	5	FJ236030.1	species	not analyzed	unidentified
S110	Food leftover 16	Food leftovers	*Pleurotus ostreatus*<br>*P. citrinopileatus*<br>*+ other species of Pleurotus*	99-100%	>5<br>1<br>po kilka	AY450345.1<br>JF736661.1	genus	not analyzed	unidentified
S111	Food leftover 17	Food leftovers	*Agaricus bisporus*	99%	5	AY484692.1	species[3]	not analyzed	unidentified
S112	Food leftover 18	Food leftovers	*Agaricus bisporus*	99%	5	AY484692.1	species[3]	not analyzed	unidentified
S126	Food leftover 21	Food leftovers	*Agaricus bisporus*	99%	5	AY484692.1	species[3]	not analyzed	unidentified
S127	Food leftover 22	Food leftovers	*Agaricus bisporus*	99%	5	AY484692.1	species[3]	not analyzed	unidentified
S128	Food leftover 23	Food leftovers	*Agaricus bisporus*	99%	5	AY484692.1	species[3]	not analyzed	unidentified
S130	Food leftover 25	Food leftovers	*Agaricus bisporus*	99%	5	AY484692.1	species[3]	not analyzed	unidentified
S131	Food leftover 26	Food leftovers	*Amanita muscaria*	97-98%	>5	JQ685713.1	species[9]	not analyzed	unidentified
S132	Food leftover 27	Food leftovers	*Amanita rubescens*	99%	1	AJ889923.1	species	not analyzed	unidentified
S133	Food leftover 28	Food leftovers	*Psilocybe semilanceata*	99%	>5	HM035080.1	species[2]	not analyzed	unidentified
S134	Food leftover 29	Food leftovers	*Marasmius alliaceus*	99%	5	JN943597.1	species	not analyzed	unidentified
S135	Food leftover 30	Food leftovers	*Kuehneromyces mutabilis*	99%	1	EU029944.1	species	not analyzed	unidentified
S136	Food leftover 31	Food leftovers	*Russula* spp.	95-98%	>5	HQ604846.1	genus	not analyzed	unidentified
S137	Food leftover 32	Food leftovers	*Russula* spp.	94-95%	>5	DQ367913.1	genus	not analyzed	unidentified
S138	Food leftover 33	Food leftovers	*Boletus calopus*	100%	3	HM347645.1	species	not analyzed	unidentified
S139	Food leftover 34	Food leftovers	*Boletus luridiformis*[7]	100%	3	FM958177.1	species	not analyzed	unidentified
S140	Food leftover 35	Food leftovers	*Hypholoma fasciculare*	100%	5	FJ430717.1	species	not analyzed	unidentified
S146	Food leftover 36	Food leftovers	*Xerocomus badius*	97%	>5	HQ207696.1	species	not analyzed	unidentified
S147	Food leftover 37	Food leftovers	*Stropharia rugosoannulata*	99%	1	KC176328.1	species	not analyzed	unidentified
S149	Food leftover 39	Food leftovers	*Stropharia rugosoannulata*	99%	1	KC176328.1	species	not analyzed	unidentified
S152	Food leftover 40	Food leftovers	*Chlorophyllum brunneum*	99-100%	>5	AY083208.1	species	not analyzed	unidentified
S159	Food leftover 41	Food leftovers	*Chlorophyllum brunneum*	99-100%	>5	AY083208.1	species	no spores	unidentified
S164	Food leftover 42	Food leftovers	*Leccinum versipelle*	99-100%	>5	AY538831.1	species[12]	not analyzed	unidentified
S165	Food leftover 42	Food leftovers	*Leccinum versipelle*	99-100%	>5	AY538831.1	species[12]	not analyzed	unidentified
S166	Food leftover 43	Food leftovers	*Russula spp.*<br>*(max. R. decolorans*<br>*R. cf. vinosa)*	93-95%	>5	DQ367913.1<br>FJ845428.1	genus	*Amanita citrina*	species
S167	Food leftover 43	Food leftovers	*Russula spp.*<br>*(max. R. decolorans*<br>*R. cf. vinosa)*	93-95%	>5	DQ367913.1<br>FJ845428.1	genus	*Amanita citrina*	species
S168	Food leftover 43	Food leftovers	*Russula vinosa*	99-100%	3	AF418638.1	species[8]	*Amanita citrina*	species

S186	Food leftover 44	Food leftovers	*Amanita muscaria*	99-100%	>5	JF899546.1	species[9]	*Amanita muscaria*	species
S187	Food leftover 44	Food leftovers	*Amanita muscaria*	99-100%	>5	JF899546.1	species[9]	*Amanita muscaria*	species
S188	Food leftover 44	Food leftovers	*Amanita muscaria*	99-100%	>5	JF899546.1	species[9]	*Amanita muscaria*	species
S201	Food leftover 45	Food leftovers	no sequence	-			unidentified	*Panaeolus papilionaceus*	species
S202	Food leftover 46	Food leftovers	*Amanita muscaria*	99-100%	>5	JF899546.1	species[9]	not analyzed	unidentified
S203	Food leftover 46	Food leftovers	*Amanita muscaria*	99-100%	>5	JF899546.1	species[9]	not analyzed	unidentified
S005	Patient 05	Gastric contents with mushroom fragments	*Bovista aestivalis*	98-99%	3	DQ112620.1	species[13]	not analyzed	unidentified
S008	Patient 08	Gastric contents with mushroom fragments	no sequence	-			unidentified	*Macrolepiota procera*	species
S010	Patient 10	Gastric contents with mushroom fragments	no sequence	-			unidentified	no spores	unidentified
S011	Patient 09	Gastric contents with mushroom fragments	*Leccinum* spp.<br>(max. *L. aurantiacum*)	98-100%	>5	AF454569.1	genus	not analyzed	unidentified
S036	Patient 11	Gastric contents with mushroom fragments	*Tricholoma sulphureum*	99-100%	3	AY462037.1	species[14]	*Tricholoma sp.*<br>*Boletaceae*	genus family
S042	Patient 12	Gastric contents with mushroom fragments	*Cortinarius cf. sertipes*<br>*C. velenovskyi*<br>*C. candelaris*	98-99%	1<br>1<br>1	AJ889969.1<br>GQ159907.1<br>GQ159883.1	genus	*Inocybe erubescens*	species
S045	Patient 14	Gastric contents with mushroom fragments	*Lactarius rufus*	99-100%	>5	DQ097868.1	species	*Gyromitra esculenta*	species
S047	Patient 15	Gastric contents with mushroom fragments	*Chlorophyllum brunneum*	99-100%	>5	AY083208.1	species	not analyzed	unidentified
S048	Patient 16	Gastric contents with mushroom fragments	*Chlorophyllum brunneum*	99-100%	>5	AY081222.1	species	not analyzed	unidentified
S049	Patient 17	Gastric contents with mushroom fragments	*Agaricus xanthodermus*	100%	2	AY484689.1	species	not analyzed	unidentified
S050	Patient 12	Gastric contents with mushroom fragments	*Agaricus xanthodermus*	99%	2	AY484689.1	species	no spores	unidentified
S051	Patient 13	Gastric contents with mushroom fragments	*Tylopilus felleus*	99-100%	4	HM190016.1	species	no spores	unidentified
S053	Patient 15	Gastric contents with mushroom fragments	*Lactarius rufus*	99-100%	>5	DQ097868.1	species	*Amanita muscaria*	species
S055	Patient 17	Gastric contents with mushroom fragments	*Scleroderma areolatum*<br>*S. verrucosum*	99%	>5<br>1	FM213352.1<br>EU784416.1	species group	*Scleroderma sp.*<br>*Boletaceae*	genus family
S062	Patient 20	Gastric contents with mushroom fragments	*Macrolepiota procera*	100%	2	JQ683106.1	species[6]	not analyzed	unidentified
S063	Patient 20	Gastric contents with mushroom fragments	*Macrolepiota procera*	100%	2	JQ683106.1	species[6]	not analyzed	unidentified
S064	Patient 21	Gastric contents with mushroom fragments	*Suillus luteus*	99-100%	5	FJ236030.1	species	no spores	unidentified
S066	Patient 22	Gastric contents with mushroom fragments	*Lactarius rufus*	99-100%	>5	DQ097868.1	species	*Russula sp.*	genus
S078	Patient 24	Gastric contents with mushroom fragments	*Scleroderma bovista*	99%	>5	JX030276.1	species	not analyzed	unidentified
S080	Patient 25	Gastric contents with mushroom fragments	*Suillus bovinus*	100%	1	AY898623.1	species	not analyzed	unidentified
S082	Patient 27	Gastric contents with mushroom fragments	*Xerocomus badius*	100%	>5	HQ207696.1	species	not analyzed	unidentified
S083	Patient 27	Gastric contents with mushroom fragments	*Xerocomus badius*	99%	>5	HQ207696.1	species	not analyzed	unidentified
S084	Patient 28	Gastric contents with mushroom fragments	*Xerocomus badius*	100%	>5	HQ207696.1	species	not analyzed	unidentified
S085	Patient 29	Gastric contents with mushroom fragments	*Echinoderma aspera*	100%	1	KC581336.1	species	not analyzed	unidentified
S088	Patient 30	Gastric contents with mushroom fragments	*Piptoporus betulinus*	97-98%	2	JQ700297.1	species	not analyzed	unidentified

S090	Patient 31	Gastric contents with mushroom fragments	*Phlebia rufa*	99%	3	AF141628.1	species[15]	not analyzed	unidentified
S091	Patient 32	Gastric contents with mushroom fragments	*Xerocomus badius*	100%	>5	HQ207696.1	species	not analyzed	unidentified
S092	Patient 32	Gastric contents with mushroom fragments	*Xerocomus badius*	100%	>5	HQ207696.1	species	not analyzed	unidentified
S094	Patient 33	Gastric contents with mushroom fragments	*Bjerkandera adusta*	99%	>5	KC176354.1	species	not analyzed	unidentified
S099	Patient 37	Gastric contents with mushroom fragments	*Xerocomus badius*	100%	>5	HQ207696.1	species	not analyzed	unidentified
S105	Patient 40	Gastric contents with mushroom fragments	*Armillaria spp.* *(max. A. sinapina* *A. calvescens* *A. gallica)*	96%	wszystkie >5	AY213565.1 KC176342.1 AY213570.1	genus[16]	not analyzed	unidentified
S151	Patient 54	Gastric contents with mushroom fragments	*Macrolepiota procera*	99%	4	JQ683106.1	species	*Macrolepiota procera*	species
S153	Patient 55	Gastric contents with mushroom fragments	*Kwoniella europaea17* *K. mangrovensis* *K. botswanensis*	99-100%	4 >5 >5	HE984340.1 HE996973.1 HF545756.1	genus	not analyzed	unidentified
S155	Patient 57	Gastric contents with mushroom fragments	*Boletus calopus*	100%	3	HM347645.1	species	*Boletaceae*	family
S156	Patient 58	Gastric contents with mushroom fragments	no sequence	-			unidentified	*Macrolepiota procera*	species
S157	Patient 59	Gastric contents with mushroom fragments	*Boletus luridiformis*[7]	98-100%	>5	FM958177.1	species	no spores	unidentified
S163	Patient 63	Gastric contents with mushroom fragments	*Bjerkandera adusta*	98-99%	>5	FJ608590.1	species	not analyzed	unidentified
S169	Patient 64	Gastric contents with mushroom fragments	*Amanita crocea* *A.velosa18*	96-97%	1 2	JQ912665.1 GQ250409.1	genus	*Amanita citrina*	species
S170	Patient 65	Gastric contents with mushroom fragments	no sequence	-			unidentified	*Russula sp.*	genus
S171	Patient 65	Gastric contents with mushroom fragments	*Boletus luridiformis*[7]	98-99%	5	DQ131633.1	species	no spores	unidentified
S174	Patient 66	Gastric contents with mushroom fragments	*Macrolepiota fuliginosa*	99%	1	AF482841.1	species	*Macrolepiota procera* *Boletaceae*	species family
S175	Patient 67	Gastric contents with mushroom fragments	*Chlorophyllum olivieri* *Macrolepiota procera*	99-100%	1 1	AY081242.1 JQ683127.1	genus group[19]	*Macrolepiota procera* *Boletaceae*	species family
S177	Patient 68	Gastric contents with mushroom fragments	*Agaricus spp.* *(max. A. abruptibulbus* *A. sylvaticus)*	97%	>5	AY484673.1 AF059219.1	genus[20]	not analyzed	unidentified
S181	Patient 70	Gastric contents with mushroom fragments	*Chlorophyllum brunneum*	99-100%	>5	AY083208.1	species	*Macrolepiota procera*	species
S184	Patient 72	Gastric contents with mushroom fragments	*Hygrophoropsis aurantiaca*	99-100%	4	HF951531.1	species	*Hygrophoropsis aurantiaca*	species
S185	Patient 73	Gastric contents with mushroom fragments	*Russula vinosa*	99%	3	AF418638.1	species[8]	not analyzed	unidentified
S190	Patient 75	Gastric contents with mushroom fragments	*Macrolepiota procera*	100%	2	JQ683106.1	species[6]	*Macrolepiota procera*	species
S191	Patient 75	Gastric contents with mushroom fragments	*Russula vinosa*	97%	1	AF418638.1	species	*Macrolepiota procera*	species
S195	Patient 77	Gastric contents with mushroom fragments	*Amanita muscaria*	99-100%	>5	JF899546.1	species[9]	no spores	unidentified
S199	Patient 81	Gastric contents with mushroom fragments	*Stropharia rugosoannulata*	97%	1	KC176328.1	species	no spores	unidentified
S200	Patient 82	Gastric contents with mushroom fragments	*Boletus calopus*	100%	3	HM347645.1	species	*Macrolepiota procera*	species
S205	Patient 82	Gastric contents with mushroom fragments	*Amanita muscaria*	99-100%	>5	JF899546.1	species[9]	not analyzed	unidentified
S214	Patient 88	Gastric contents with mushroom fragments	*Chlorophyllum olivieri* *Macrolepiota procera*	99-100%	1 1	AY081242.1 JQ683127.1	genus group[19]	*Macrolepiota procera*	species
S215	Patient 88	Gastric contents with	*Chlorophyllum olivieri* *Macrolepiota procera*	99-100%	1	AY081242.1	genus group[19]	not analyzed	unidentified

S216	Patient 89	Gastric contents with mushroom fragments	*Chlorophyllum olivieri*<br>*Macrolepiota procera*	99-100%	3<br>1	AY081242.1<br>JQ683127.1	genus group[19]	not analyzed	unidentified
S217	Patient 90	Gastric contents with mushroom fragments	*Amanita muscaria*	99-100%	>5	JF899546.1	species[9]	not analyzed	unidentified
S220	Patient 93	Gastric contents with mushroom fragments	*Xerocomus badius*	99-100%	>5	HQ207696.1	species	not analyzed	unidentified
S004	Patient 04	Gastric contents without mushroom fragments	*Russula cyanoxantha*	99-100%	>5	AF418608.1	species	not analyzed	unidentified
S118	Patient 46	Gastric contents without mushroom fragments	*Macrolepiota procera*	99%	1	JQ683109.1	species	not analyzed	unidentified
S120	Patient 48	Gastric contents without mushroom fragments	*Boletus calopus*	100%	3	HM347645.1	species	not analyzed	unidentified
S121	Patient 49	Gastric contents without mushroom fragments	*Armillaria ostoyae*	99%	>5	AY213552.1	species[21]	not analyzed	unidentified
S154	Patient 56	Gastric contents without mushroom fragments	*Boletus luridiformis*[7]	98-100%	>5	FM958177.1	species	*Mycena sp.*<br>*Coprinus sp.*	genus
S158	Patient 56	Gastric contents without mushroom fragments	*Boletus luridiformis*[7]	98-100%	>5	FM958177.1	species	no spores	unidentified
S160	Patient 60	Gastric contents without mushroom fragments	*Boletus luridiformis*[7]	98-99%	>5	DQ131633.1	species	no spores	unidentified
S162	Patient 62	Gastric contents without mushroom fragments	*Macrolepiota procera*	100%	2	JQ683106.1	species[6]	no spores	unidentified
S197	Patient 79	Gastric contents without mushroom fragments	*Amanita muscaria*	99-100%	>5	JF899546.1	species[9]	not analyzed	unidentified
S204	Patient 81	Gastric contents without mushroom fragments	*Amanita muscaria*	99-100%	>5	JF899546.1	species[9]	no spores	unidentified
S209	Patient 85	Gastric contents without mushroom fragments	*Amanita muscaria*	99%	>5	JF899546.1	species[9]	not analyzed	unidentified

1) Sequences that match at the level of 99-100% also named *A. subpediades* (JN684790.1), *A. semiorbicularis* (AY194536.1), and *A.arenicola* (JN684805.1), which are synonyms of *A. pediades*.
2) Match at the level of 99-100% also with an individual sequence of *P. fasciata* (AB158635.1) – an Asian species occurring in China and Japan.
3) Match at the level of 99% also with individual sequences: *A. subfloccosus* - AY484698.1 (European species, known in countries such as Denmark, Czech and Ukraine) and *A. subrufescens* - AY484674.1 (species known in North and South America, but also Western Europe). However, the origin of the material indicates that the most probable identity of the sample is *A. bisporus*.
4) Match at the level of 99-100% also with sequences of *L. fallax* (KC581340.1); however, this is a species occurring only in North America.
5) Match at the level of 97-98% also with sequences of *Tricholoma mongolicum* (KC413941.1); however, this is a species occurring only in Asia.
6) Match at the level of 99-100% also with sequences of the closely-related species *M. puellaris* (JQ683121.1), originating from Armenia.
7) Sequences in GenBank are labelled with the synonymous name *B. erythropus*.
8) Match at the level of 99-100% also with an individual sequence of *R. occidentalis* (AY228349.1,); however, this is a species occurring exclusively in North America.
9) Match at the level of 98-99% also with an individual sequence of *A. gemmata* (AF335440.1) originating from North America (possible error in GenBank database).
10) *A. excellens* (synonym *A. urinascens var. excellens*), and *A. nivescens* (synonym *A. osecanus*) are species occurring in Europe. *A. albolutescens* is a species occurring in North America. All the mentioned species are related and belong to one section (together with *A. sylvicola*) (Geml J, Royse DJ. Molecular phylogeny and cultivation of Agaricus species. In: Proceedings of the IV International Conferenceon Mushroom Biology and Mushroom Products; 2002 Feb 20–23; Cuernavaca, Mexico; p. 111–20.)
11) The closest sequences in GenBank are labelled with the synonymous name *L. deliciosus* var. *deterrimus*.
12) Sequences match at the level of 99-100%, also labelled with synonymous names *L. callitrichum* (AY538829.1), *L. percandidum* (AY538828.1), and *L. roseotinctum* (AY538826.1).
13) Match at the level of 99%, also with an individual sequence of *B. promontorii* (DQ112621.1); however, this is a species occurring exclusively in South Africa.
14) Match at the level of 99%, also with an individual sequence of *T. bufonium* (AY462030.1), which is a species that is very closely related to *T. sulphureum*. According to the work of Comandini et al. (Comandini O, Haug I, Rinaldi AC, Kuyper TW. Uniting Tricholoma sulphureum and T. bufonium. Mycol Res. 2004;108:1162-71. Medline:15535067 doi:10.1017/S095375620400084X), they are synonyms.
15) Sequences match at the level of 99% also labelled with the synonymous name *Phlebia acerina* (AB210080.1).
16) *A. sinapina, A. calvescens* – species occurring in North America, *A. lutea* (synonym *Armiallaria gallica*) – known species occurring in Europe and North America
17) Genus *Kwoniella* encompasses five currently known species of mushrooms from the order Tremellales, closely related to the genus *Filobasidiella.* These are saprobiontic microscopic fungi that do not form fruiting bodies (41,42,43,44).
18) *Amanita velosa* is a species occurring in North America.
19) Match at the level of 99% also with an individual sequence of *Morchella esculenta* (JQ691498.1) – evidently erroneously labelled sequence.
20) Match at the level of 99-100% also with sequences of *Agaricus californicus* (e.g. FJ755229.1); however, it is a species that is known from North America.
21) Match at the level of 99% also with sequences of species: *A. gemina* (AY213556.1), *A. sinapina* (AY213565.1) and *A. calvescens* (AY213565.1); however, these are species occurring exclusively in North America.
